# Mechanical Characterization on Solvent Treated Cellulose Nanofiber Preforms Using Solution Dipping–Hot Press Technique

**DOI:** 10.3390/nano10050841

**Published:** 2020-04-29

**Authors:** Devendran Thirunavukarasu, Yoshinobu Shimamura, Keiichiro Tohgo, Tomoyuki Fujii

**Affiliations:** 1 Department of Environment and Energy System, Graduate School of Science and Technology, Shizuoka University, 3-5-1 Johoku, Naka-ku, Hamamatsu, Shizuoka 432-8561, Japan; 2 Department of Mechanical Engineering, Shizuoka University, 3-5-1 Johoku, Naka-ku, Hamamatsu, Shizuoka 432-8561, Japan; shimamura.yoshinobu@shizuoka.ac.jp (Y.S.); tohgo.keiichiro@shizuoka.ac.jp (K.T.); fujii.tomoyuki@shizuoka.ac.jp (T.F.)

**Keywords:** nanocomposites, cellulose nanofibers, mechanical properties, microstructure characterization

## Abstract

Nanocomposites films were prepared by impregnating the solvent treated cellulose nanofiber (SCNF) preforms with epoxy resin using a solution dipping–hot press technique. We investigated the effect of SCNF preforms porosity on the amount of impregnated resin and tensile properties of the corresponding nanocomposites films. The porosity of the CNF preforms was successfully controlled using the solvent exchange with varying CNF concentration. The impregnated resin amount increased as the SCNF preforms porosity increased, respectively. Resulting nanocomposite films showed higher mechanical properties than that of the SCNF preforms. The best mechanical properties of composites were found with the combination of 1 wt % SCNF preform and low viscosity epoxy, exhibiting tensile strength and Young’s modulus of 77 MPa and 4.8 GPa, respectively. The composite also showed high fiber volume fraction of more than 60%.

## 1. Introduction

Depleting crude oil-based resources and environmental concerns such as global warming has generated significant interest in producing environmentally friendly materials such as biomass materials, including cellulose, hemicelluloses, and lignin [1,2,3]. Woods and plants constitute the rich sources of cellulose fibrils in the cell walls. Plant-based materials have multiple benefits, such as renewability, biodegradability, and ecofriendliness. It can be used as suitable substitutes for petroleum-based products to solve environmental problems. It can also be used in various applications including automotive, building materials, and furniture.

Recently, cellulose has been used to produce nanosized materials such as cellulose nanomaterials. Cellulose nanomaterials are derived from wood, agriculture, and their byproducts. They are categorized into cellulose nanofibers (CNFs) and cellulose nanocrystals. CNF is a long, versatile, interlocking network with a diameter of about 1–100 nm composed of crystalline and amorphous domains [4]. CNF is also called microfibrils of cellulose, which are up to several micrometers in length. Crystalline cellulose’s strength/weight ratio is much higher than that of most of structural metals; hence, it has excellent mechanical properties. CNF can be extracted from cellulosic materials through a broad variety of processes including chemical processes [5,6,7] and mechanical process [8]. Mechanical are processes widely used to defibrillate cellulosic fibers into CNF, which includes drying, homogenization by high pressure, microfluidization, grinding, cryocrushing, ultrasonic high strength, steam explosion, and electrospinning. The aqueous counter collision method (ACC) developed by Kondo et al. [8] is one of the mechanical process methods of CNF isolation, and is suitable for CNF mass production. The ACC method requires the use of dual high-speed water jets to cleave interfacial interactions between cellulose molecules and also disintegrates native fibers into CNFs without chemical modification, which in fact exposes the inherent structural difference of the raw materials [9].

The preparation of CNF thermoset nanocomposites is accomplished by various manufacturing methods such as impregnation of resin, solution casting, melting process, and vacuum infusion [10,11,12,13]. Chirayil et al. [14,15] incorporated the cellulose nanofibrils (CNF) into unsaturated polyester resin (UPR) by conventional mechanical mixing and compression molding process. The CNF/UPR mixture was poured onto the mold and then cured at room temperature for 12 h under constant 1 MPa pressure. The mechanical properties of CNF/UPR composites were improved at the lower CNF content. The Young’s modulus and tensile strength of the 1 wt % CNF reinforced composites were 2.03 GPa and 51.6 MPa, respectively. Ansari et al. [16,17] reported the high-volume fractions of composites made of cellulose nanofiber (CNF) by impregnating a wet porous CNF network with an acetone/epoxy/amine solution. Microfibrillated cellulose was dispersed in silane and titanate coupling agents in acetone were reported by Lu et al. [18]. This suspension was transferred to a Teflon petri dish and dried in an oven at 30 °C, the storage modulus increased from 2.59 to 3.45 GPa at 5 wt % of CNF. Tang and Weder [19] suggested a different approach by avoiding coupling agents; they dispersed unmodified cellulose whiskers with a high aspect ratio in dimethyl formamide (DMF) using the freeze-drying or solvent exchange method. The nanocomposite films were prepared by mixing epoxy and amine components, later casted and dried by elevated curing temperature. At 30 °C, the storage modulus of the neat epoxy was 1.6 GPa and the highest modulus of 5.7 GPa were obtained at 20 vol% of tunicate whiskers. Shibata and Nakai [20] dispersed the microfibrillated cellulose (MFC) into biobased epoxy resin, then freeze-dried the suspension, and cured at high temperature. The Young’s modulus increased for 15 wt % microfibrillated cellulose content from 1.7 to 2.6 GPa, and the tensile strength increased from 60 to around 80 MPa for 10 wt % MFC. Nakagaito et al. [21] prepared a nanocomposite composed of microfibrillated cellulose and phenol resin. They reported an elastic modulus of 12 GPa at 40 wt % cellulose content, corresponding closely to the values of glass fibers. Tuuka et al. [22] impregnated the bio epoxy resin into the porous cellulose nanofiber network using ice-templating and vacuum infusion process. The Young’s modulus and tensile strength of the nanocomposites were 1.52 GPa and 66 MPa with 13% volume fraction. Stachewicz et al. [23] reported that the electrospun nonwoven PA6 nanofiber mats/polyvinyl alcohol (PVA) composites were prepared by using solution-based processing method. The nanocomposites were stated to have the highest failure strength for 16 wt % of PVA matrix. In previous methods, wet CNF preforms were impregnated into the resin system. It was difficult to impregnate the resin, completely and also continuous production of CNF/epoxy composites was not possible.

In the present study, ACC nanopulverized CNF of 0.5 wt %, 1 wt %, and 1.5 wt % concentration was used. CNF preforms were fabricated by filtering the well dispersed CNF suspension to obtain the gel cake. Second, CNF gel cakes were solvent exchanged into acetone and subsequently dried in a hot press to obtain the solvent treated CNF preforms. Previously, we reported that the density and porosity of CNF preforms were controlled by varying the concentration of cellulose nanofibers as a consequence of the solvent exchange [24]. The main novelty of this study is to impregnate the epoxy resin into the dried porous SCNF preforms to produce the cellulose nanofiber reinforced composites films using a solution dipping–hot press technique. This technique is easy to scale up and is a cost-effective process. The viscosity of the impregnation solutions was adjusted to vary the diluent and hardener amount of the resin into the CNF sheets. The effects on the mechanical properties and morphological characterization of the resulting CNF/epoxy composite films were also investigated.

## 2. Materials and Methods 

### 2.1. Materials 

Nanopulverized cellulose nanofiber were prepared by an aqueous counter collision method from Sugino machine Ltd., Toyama, Japan. The concentration and viscosity of CNF was 11 wt % and 3000 mPa∙s, respectively. Bisphenol-A type (jER 828) epoxy resin, modified aliphatic amine grade curing agent and alkyl monoglycidyl ether (YED 111 N) diluent were purchased from Mitsubishi chemical, Tokyo, Japan. The viscosity of resin 828, hardener, diluent: 12–15 Pa∙s, 0.2–0.5 Pa#x2219;s, 0.0065–0.0075 Pa∙s. The curing conditions of epoxy resin system were 23 °C for 24 h and then at 80 °C for 3 h. Laboratory grade acetone were purchased from Wako chemicals, Osaka, Japan.

**Figure 1:**
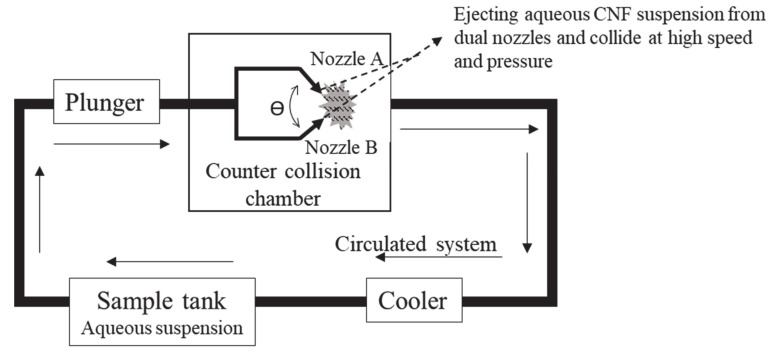
Illustration of the aqueous counter collision (ACC) method.

The aqueous counter collision system developed by the Sugino Machine Co., Ltd. Toyama, Japan was adapted in this study. Making naturally occurring cellulose fibers into nanofibers solely by the use of aqueous counter collision (ACC) as shown in Figure 1, without any chemical modification was reported by Kondo et al. [8]. In this process, equivalent aqueous suspensions of cellulose are ejected from dual nozzles and collide at high speed and pressure. Even a few repetitions of the collision process are sufficient to produce nanosized fibers dispersed in water. In this result, CNF with a small diameter approximately 10–50 nm and a few micrometers of fiber length were obtained. ACC nanopulverized CNF was not involved in any chemical treatment, purely it is a sustainable nanofiber.

### 2.2. CNF Preform Preparation

Gel like-CNF preform was formed by first diluting 11 wt % nanocellulose suspension to 0.5, 1, and 1.5 wt % concentrations. CNF suspension (100 mL) was then stirred with a magnetic stirrer at 1500 rpm for 15 min and filtered through a Buchner funnel with a PTFE filter (diameter 90 mm/pore size 0.22 μm). After vacuum filtration, the wet CNF preform was immersed in acetone for 3 h, and then the solvent was changed by new batch of solvent. This exchange process was repeated four times for complete substitution of water with the solvent and then the solvent treated CNF preform was dried in a hot press at 105 °C for 10 min under 0.5 MPa pressure. The resulted solvent treated CNF (SCNF) preforms and water dried CNF (WCNF) of different porosities and thicknesses were obtained.

**Figure 2:**
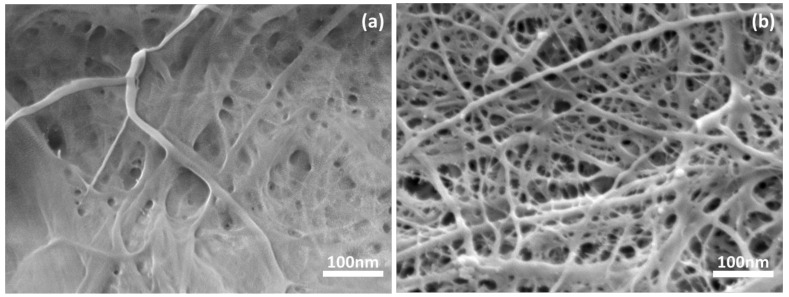
FESEM images of (**a**) water-dried cellulose nanofiber (CNF) preform and (**b**) solvent treated CNF preform.

**Figure 3:**
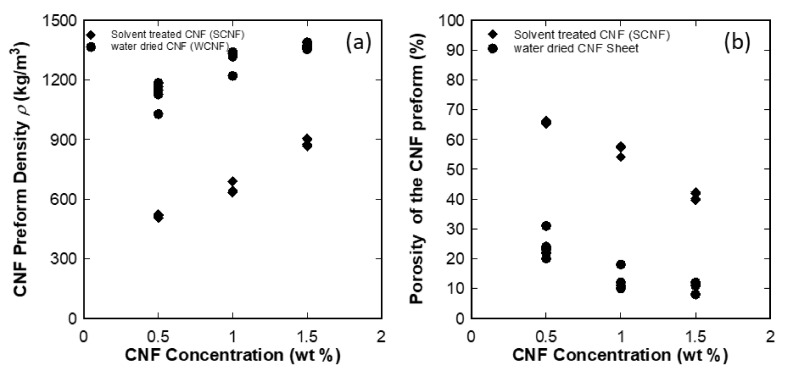
(**a**) Density and (**b**) porosity of the CNF preform as a function of CNF concentration in wt %.

The typical morphological characteristics of prepared CNF preforms are shown in Figure 2. The FE-SEM images revealed that the solvent exchange method resulted in highly porous CNF network structure, and it would be suitable for composite fabrication as shown in Figure 3b, while the water-dried method yielded more densified and less porous microstructure as shown in Figure 3a [24].

Figure 3 and Table S1 shows the density and porosity of the CNF preforms as a function of CNF concentration. The results revealed that the density and porosity were successfully controlled using the solvent exchange with different CNF concentration. The solvent exchange method may weaken the interaction force between CNFs when water is replaced by acetone. The less hydrophilic character of acetone decreases the capillary effects at the time of solvent exchange [25]. While, acetone evaporates from the CNF sheet; assumed that few hydrogen bonds break and it leads to low nanofibers compaction with high porosity. The solvent treated CNF preforms prepared by the solvent exchange–hot press technique had the modulus of 2.1–2.2 GPa and the strength of 21–29 MPa respectively with a different concentration of cellulose nanofibers suspensions [24].

### 2.3. Impregnation Solution Preparation

Impregnation solutions with different viscosity were prepared by varying the ratio of epoxy resin, hardener, and diluent to achieve the desired viscosity for impregnation. The impregnation solution ratio is as shown in Table 1. The impregnation solutions were mixed homogenously at room temperature. The viscosity of the impregnation solution was measured using an AMETEK Brookfield DV-II, Middleboro, MA rotational type viscometer.

### 2.4. Preparation of CNF/Epoxy Nanocomposite Films

**Figure 4:**
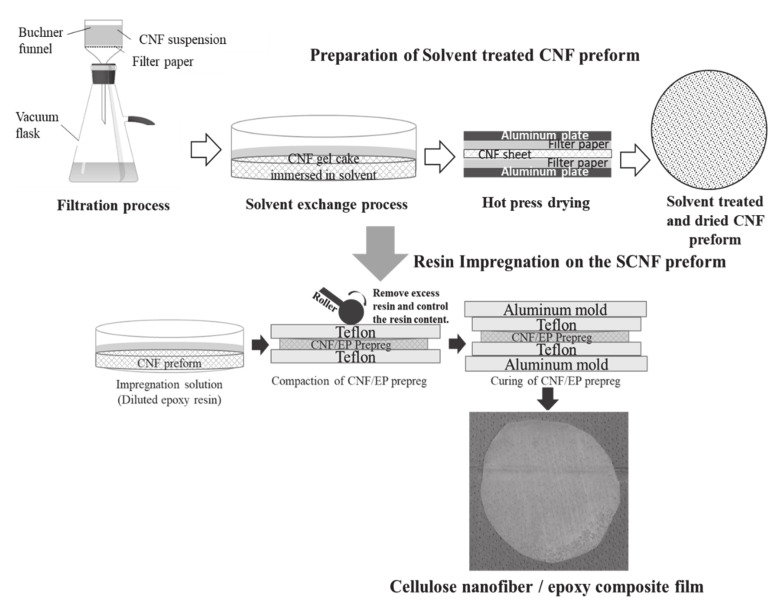
Preparation of the solvent treated CNF/epoxy nanocomposite films.

The preparation technique of nanocomposite films is as shown in Figure 4. The solvent treated cellulose nanofiber (SCNF) preforms were dipped in the diluted epoxy resin solution at different time intervals, then resin impregnated SCNF films were dried in room temperature for 24 h. Later, films were cured in hot press for 3 h at 80 °C with 1 MPa pressure. For the reference, we prepared the water dried CNF/epoxy composite films also. Water dried CNF (WCNF) preforms were prepared without a solvent exchange process and dried in the hot press at 105 °C for 10 minutes under 0.5 MPa pressure. The resulting nanocomposite film thicknesses were varying from 95 to 655 µm. The obtained nanocomposite films were characterized by FE-SEM to observe the impregnation condition.

## 3. Results

### 3.1. Resin Impregantion of Solvent Treated CNF Preforms

The porosity of solvent treated CNF preforms was successfully controlled by the solvent exchange method and vacuum filtration process with varying concentrations of CNF. Furthermore, nanocomposite films were produced by impregnating the solvent treated CNF preforms with diluted epoxy resin system using the solution dipping technique. The different concentration of CNF was formed by a network of nanofibers. The CNF network has a low density and relatively high porosities, which is a significant fact considering the volume fraction of the nanocomposite films. These networks have also been resin impregnated with low viscosity impregnation resin to form strong composite materials. The impregnated resin amount increased as the SCNF preform density decreased, respectively. The best improvements were achieved with 60 vol.% CNF contents in the nanocomposite films. This was suggested to be a result of better fiber dispersion and strong adhesion to the matrix. The mechanical properties of the prepared CNF nanocomposite films were found to improve as a function of increased fiber volume fraction as discussed in Section 4.1. However, the volume fraction was influenced by the porosities and CNF content of the nanocomposite films. Significantly, the presented fabrication method allowed us to control the impregnation resin content as well as decrease the processing time.

### 3.2. Measurement of Physical Properties of CNF Epoxy Nanocomposites

The density, volume fraction, and impregnated resin content of the composites were calculated by using the rule of mixtures.

$$\mathrm{Density},\;\rho =\frac{\mathrm{Mass}\;\left(g\right)}{{\mathrm{Volume}\;(\mathrm{cm}}^{3})}\;\mathrm{Volume}\;\mathrm{fraction}{,\;V}_{f}=\frac{W_{f}{\;\text{×}\;\rho }_{c}}{\rho_{f}}\;\;\mathrm{Weight}\;\mathrm{fraction}{,\;W}_{f\;}=1\;-{\;W}_{m}\;\mathrm{Impregnated}\;\mathrm{resin}\;\mathrm{content}=\frac{w_{2\;}-{\;w}_{1}}{w_{1}}\times \;100\%$$

where V_f_ is fiber volume fraction of the composites, ρ_c_ is the composites density and ρ_f_ is the CNF preform density, W_f_ and W_m_ are the weight fraction of CNF and matrix, respectively, and w_1_ and w_2_ are the weight of the CNF preform and nanocomposites, respectively.

**Figure 5:**
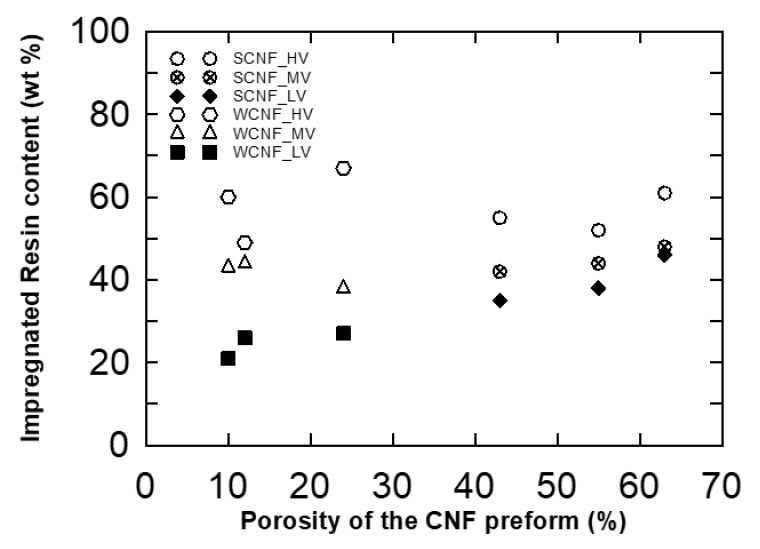
Relationships between CNF preform porosity and the impregnated resin content (wt %).

The impregnation resin content as a function of the CNF preform porosities is shown in Figure 5. The results revealed that the resin content varied from 20 to 60 wt %, and there was a roughly linear relation between the porosity of the CNF preform and the resin content for low viscosity epoxy. For medium and high viscosity epoxy, deviation from the linear relation was found because the resin impregnation into preform was difficult for lower porosity and thicker resin rich region was found on both surfaces of the preform.

### 3.3. Tensile Test

Tensile tests of the CNF epoxy nanocomposite films were carried out according to JIS K 7127 on a Shimadzu Model 50 kN, Kyoto, Japan, uniaxial tensile test machine. Test samples of 25 mm in the gauge length and width 5 mm were tested at a speed rate of 0.4 mm/min under the relative humidity of 65% and temperature at 26 °C. Totally, five samples were tested for each fabrication condition. Young’s modulus was determined at the slope at 0.25–0.5% of the stress–strain curve, and the tensile strength was found to obtain maximum stress at specimen fractured.

### 3.4. Field-Emission Scanning Electron Microscope (FE-SEM) 

FE-SEM observation was conducted using Hitachi SU8010, Japan, to investigate the impregnation condition and fracture surface of the CNF epoxy nanocomposite films. To avoid the electrical charging of the sample during FE-SEM observation, the samples were mounted on aluminum stubs and then gold coated using SANYU quick coater SC-701, Tokyo, Japan. In the previous study we examined the structural morphology of CNF preforms as a consequence of solvent exchange [25]. The accelerating voltage and working distance were 3 kV and 8 mm respectively.

### 3.5. Mechanical Properties

**Figure 6:**
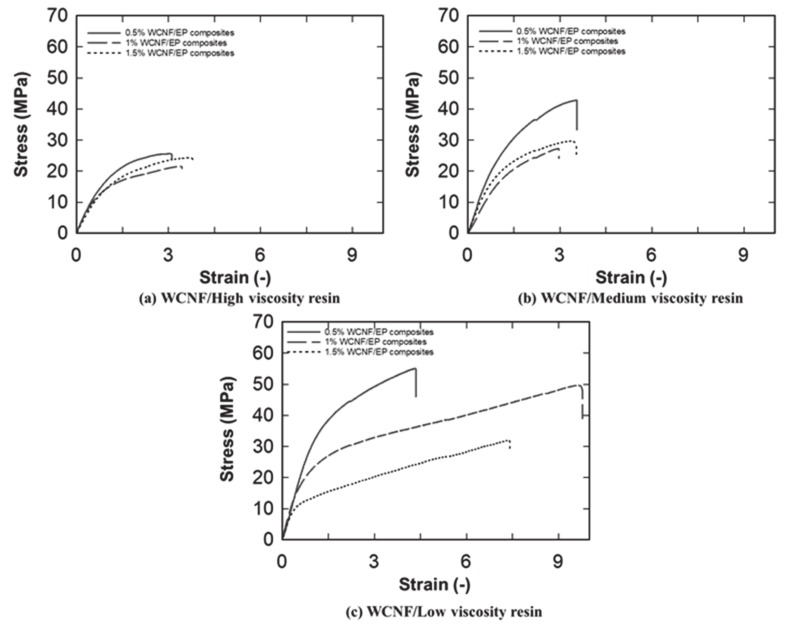
Typical stress−strain curves for (**a**) water-dried CNF/high viscosity epoxy composites; (**b**) water- dried CNF/Medium viscosity epoxy composites; (**c**) Water-dried/low viscosity epoxy composites.

**Figure 7:**
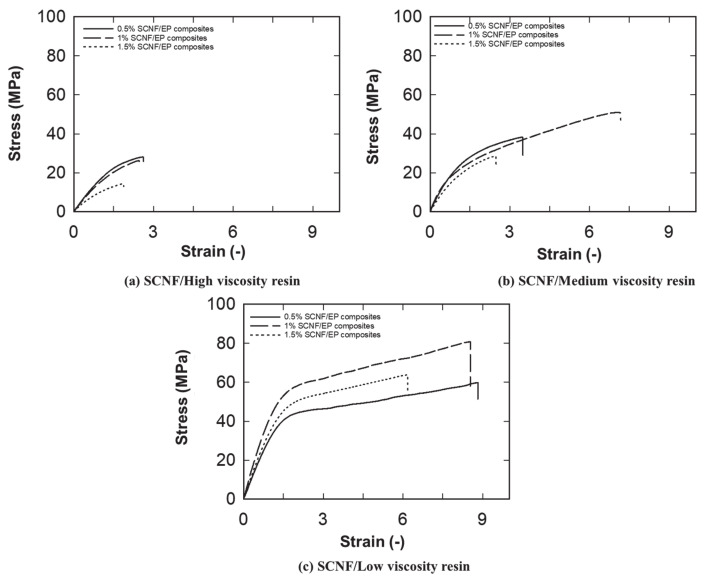
Typical stress−strain curves for (**a**) solvent treated CNF/high viscosity epoxy composites; (**b**) solvent treated CNF/medium viscosity epoxy composites; (**c**) solvent treated CNF/low viscosity epoxy composites.

Typical stress–strain curves of CNF nanocomposites films produced from solvent treated CNF preforms and water-dried CNF preforms are presented in Figure 6 and Figure 7, respectively. Stress–strain curves clearly show the reinforcing effect of the cellulose nanofibers on nanocomposites films, which is seen from the increased initial slopes of the curves and the ultimate strengths when the lower viscosity resin was used. In addition, the stress–strain behavior of CNF/epoxy nanocomposites were significantly changed while varying the viscosity of impregnation solution. This significant increase of the elongation at break was obtained while decreasing the viscosity of the impregnation solution in the nanocomposite films without the loss of stiffness.

**Figure 8:**
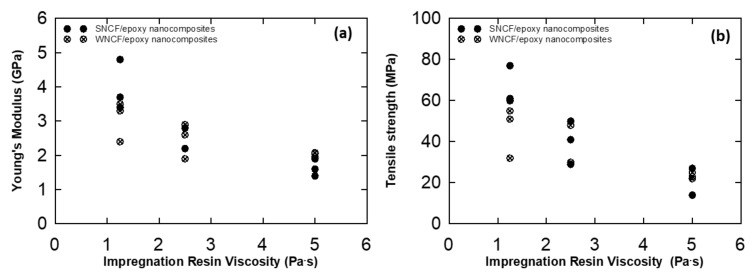
Young’s modulus (**a**) and tensile strength; (**b**) of the CNF/epoxy nanocomposite films as a function of the impregnation resin viscosity.

The Young’s modulus and tensile strength of the solvent treated CNF/epoxy and water dried CNF/epoxy nanocomposite films are summarized in Table S2 and Table S3, and are plotted as a function of resin viscosity in Figure 8. From Figure 8, it shows that the Young’s modulus and tensile strength considerably depends upon the impregnation resin viscosity. The maximum increase of the tensile strength and modulus were 77 MPa and 4.8 GPa, respectively and achieved for 1 wt % of solvent treated CNF preform with low viscous impregnation solution.

Our previous research [25] showed that the porosity of the CNF preform can be controlled by varying the concentration of aqueous cellulose nanofibers suspensions, and also by solvent exchange treatment as shown in Figure 3b. The effect of the change of the microstructure of preforms on the mechanical performance will be discussed in the discussion section.

### 3.6. Fracture Surface of CNF Epoxy Nanocomposites

**Figure 9:**
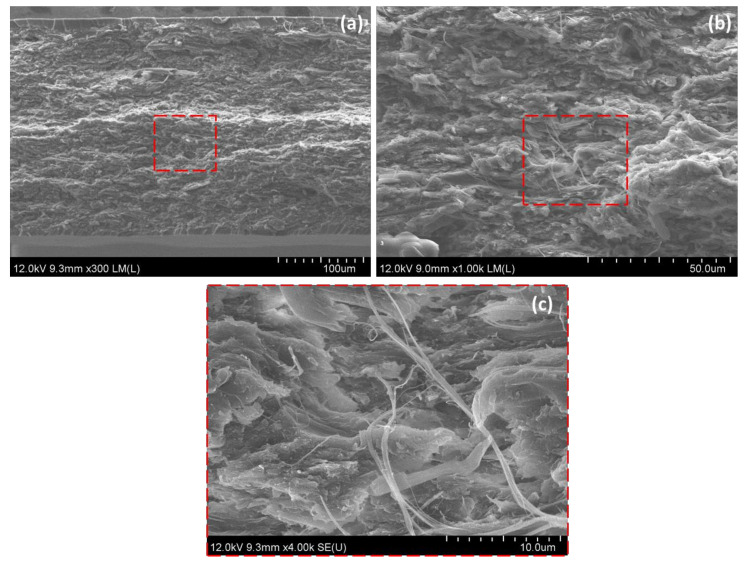
FESEM images. (**a**) Fracture surfaces of 1 wt % solvent treated CNF epoxy composites and (**b**,**c**) high magnifications of 1 wt % solvent treated CNF epoxy composites.

**Figure 10:**
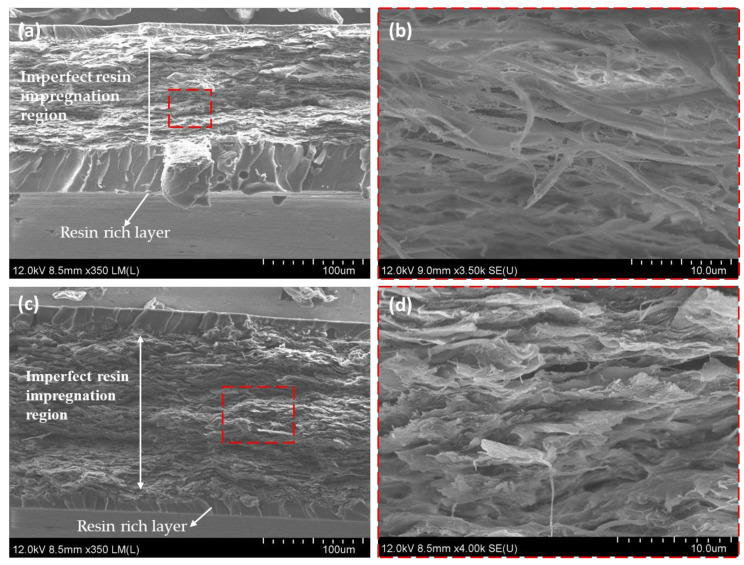
FE-SEM images of (**a**) 1 wt % water-dried CNF/epoxy composites; (**b**) high magnification of 1 wt % water-dried CNF/epoxy composites; (**c**) 1.5 wt % SCNF/epoxy composites; and (**d**) high magnification of 1.5 wt % SCNF/epoxy composites.

The impregnation conditions of nanocomposites films were observed in the FE-SEM analysis. The higher magnification images Figure 9c shows resin impregnation into SCNF preforms. It was the main reason to improve the mechanical properties of nanocomposites films. On the contrary, the FE-SEM images in Figure 10b,d for the lower porosity SCNF and water dried CNF preforms reveals that the middle region of the cross section was layered and dense fibrils microstructure, implying imperfect resin impregnation due to the low porosities of nanofibers in CNF preforms. Additionally, also, we observed the resin-rich layer in the specimen surface.

The influence of the CNF preforms porosity was based on the resin impregnation amount, the porosity was adjusted by solvent exchange of water with acetone. The resin impregnation amount increased as the porosities increased. In contrast, lower porosity SCNF and water dried CNF preform underwent nanofibers compaction during the drying process. The densities of those CNF preform were relatively high, it was the evidence of the imperfect resin impregnation of the preforms as shown in Figure 10c,d. Therefore, the optimum 1 wt % SCNF/epoxy composites had better impregnation condition than other CNF preforms. The failure mechanism of CNF/epoxy nanocomposites will be discussed in the Discussion section.

## 4. Discussion

### 4.1. Effect of Fabrication Parameters on Mechanical Properties of CNF/Epoxy Nanocomposites 

**Figure 11:**
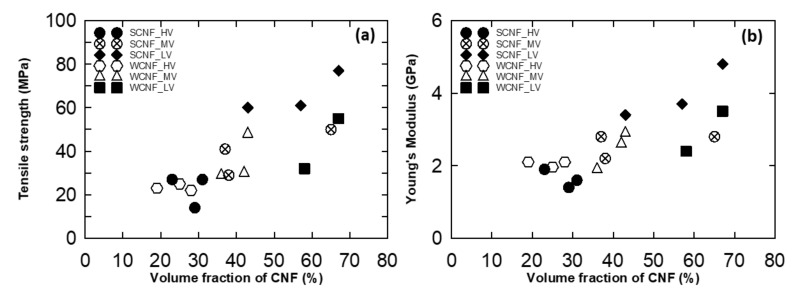
(**a**) Tensile strength vs. volume fraction of CNF in nanocomposite films and (**b**) Young’s modulus vs. volume fraction of CNF in nanocomposite films.

Resin/hardener/diluent ratios were set at high viscosity (7:2:1), medium viscosity (8:1:1), and low viscosity (7:1:2). In this study we investigated the effect of the viscosity of impregnation solutions. Figure 8 shows that lower viscosity resulted in better mechanical performance. Since low viscosity epoxy resin were easily impregnated into the CNF preforms as well as it may increase the nanofiber–epoxy interaction, it leads to enhancement in the mechanical properties of CNF epoxy nanocomposites. The relation between tensile strength, Young’s modulus and the volume fraction of CNF is shown in Figure 11. It reveals that the volume fraction of CNF primarily dominates the mechanical performance of composites as expected. Furthermore, the volume fraction of CNF was controlled by varying the impregnation solution ratio in the CNF/epoxy nanocomposites. This may be caused by the changes of viscosity as discussed above. The volume fraction of the nanofiber with different impregnated resin contents will differ, which may result in the different tensile strengths and Young’s modulus. Therefore, nanocomposites with lower viscosity resin shows higher mechanical properties than the other nanocomposite films.

From this study, we revealed that the volume fraction and mechanical properties were relatively higher than the previous studies. Additionally, also, we achieved the best mechanical properties of the 1 wt % SCNF preform with a low viscosity epoxy, it also exhibited the Young’s modulus and tensile strength of 4.8 GPa and 77 MPa with 60% of volume fraction.

In our proposed fabrication method, we dried the wet solvent treated cellulose nanofiber (SCNF) preform at 105 °C for 5–10 min under compaction pressure, followed by SCNF preforms were dipped in the diluted epoxy resin solution using the conventional prepreg manufacturing method such as the solution dipping process. The solution dipping process is an already proven method in the composite industries. It is easy to scale up and manufacture the nanocellulose/epoxy prepreg for various application like building materials, furniture, etc.

### 4.2. Failure Mechanism of CNF/Epoxy Nanocomposite Films

The FE-SEM image of the fracture surface shown in Figure 9b revealed that the pull-out of CNFs or thinner CNF bundles with a diameter of a submicrometer were rarely observed although several long pull-outs of thicker CNF bundles were found. The results imply that CNFs and thinner CNF bundles had a strong interface with epoxy, and thus were able to carry a load.

### 4.3. Stress–Strain Behavior of CNF/Epoxy Nanocomposite Films

**Figure 12:**
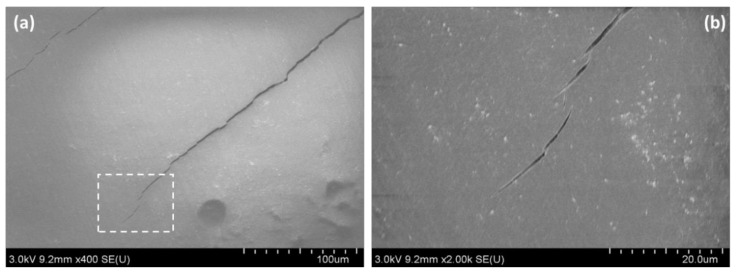
FE-SEM images of (**a**) microcracks on the 1 wt % SCNF nanocomposite surface and a (**b**) higher magnification image of microcracks on the 1 wt % SCNF nanocomposite surface.

The stress-strain curves of nanocomposite films using a low viscosity epoxy clearly shows that non-linear behavior like yielding even though epoxy used in this experiment was a brittle one. To elucidate the yield-like behavior, surface observation of a failed specimen was conducted. Figure 12 is an example of the surface observation of a failed specimen, and microcracks were found on the surface. This implies that toughening occurs by CNF, resulting in arrests of crack propagation by fiber bridging [26] at the tip of microcracks. The occurrence of microcracks caused reduction of the stiffness of specimen, resulting in the yield-like non-linear behavior.

## 5. Conclusions

Nanocomposites films were successfully produced by the novel solution dipping-hot press technique when a low viscosity epoxy of 1.25 Pa s was used. The fabrication method allowed us to control the impregnation resin content as well as decrease the processing time. The technique enabled us to fabricate composite materials with a higher fiber volume fraction of up to 60%. FE-SEM images revealed the strong interaction between CNF and epoxy. The higher volume fraction and the strong interaction was the reason for improved mechanical performance. Yield-like non-linear behavior was found for specimens with higher mechanical performance. This is probably caused by a stiffness reduction due to the occurrence of microcracks.

## Figures and Tables

**Table 1:** Proportions of impregnation solution.

Sample name	Epoxy resin (wt %)	Hardener (wt %)	Diluent (wt %)	Measured Viscosity (Pa·s)
**HV**	70	20	10	5
**MV**	80	10	10	2.5
**LV**	70	10	20	1.25

HV—high viscosity resin, MV—medium viscosity resin, LV—low viscosity resin.

## Supplementary Materials

The following are available online at https://www.mdpi.com/2079-4991/10/5/841/s1, Table S1: Physical properties of CNF preform, Table S2: Mechanical properties of water-dried CNF epoxy composites, Table S3. Mechanical properties of solvent treated CNF epoxy composites.
