# Supplementary material for: Mechanical Characterization on Solvent Treated Cellulose Nanofiber Preforms Using Solution Dipping–Hot Press Technique
Source: Nanomaterials (Basel). 2020 Apr 29;10(5):841. doi: 10.3390/nano10050841 (PMC7712792; doi:10.3390/nano10050841)
Supplement: Supplementary file 1 [file nanomaterials-10-00841-s001.pdf]

nanomaterials

Supplementary Information

Mechanical Characterization on Solvent Treated Cellulose Nanofiber Preforms Using Solution Dipping–Hot Press Technique

Devendran Thirunavukarasu [1,*], Yoshinobu Shimamura [2], Keiichiro Tohgo [2] and Tomoyuki Fujii [2]

[1] Department of Environment and Energy System, Graduate School of Science and Technology, Shizuoka University, 3–5–1 Johoku, Naka-ku, Hamamatsu, Shizuoka 432–8561, Japan

[2] Department of Mechanical Engineering, Shizuoka University, 3–5–1 Johoku, Naka-ku, Hamamatsu, Shizuoka 432–8561, Japan; shimamura.yoshinobu@shizuoka.ac.jp (Y.S.); tohgo.keiichiro@shizuoka.ac.jp (K.T.); fujii.tomoyuki@shizuoka.ac.jp (T.F.)

* Correspondence: devendran.thirunavukarasu.17@shizuoka.ac.jp; Tel.: +81-53-487-1045

Received: 21 April 2020; Accepted: 24 April 2020; Published: date

Table S1. Physical properties of CNF preform.

Sample name	CNF concentration (wt %)	Solvent exchange	Preform weight (g)	Porosity (%)	Preform density (Kg/m^3)	Thickness (μm)
**0.5 wt %SCNF**	0.5	3 h	0.51	63	553	204
**1 wt %SCNF**	1.0	3 h	1.13	55	674	321
**1.5 wt %SCNF**	1.5	3 h	1.54	43	848	335
**0.5 wt %WCNF**	0.5	-	0.53	24	1130	79
**1 wt %WCNF**	1.0	-	1.09	12	1310	152
**1.5 wt %WCNF**	1.5	-	1.48	10	1374	163

Table S2. Mechanical properties of water-dried CNF epoxy composites.

CNF concentration (wt %)	Resin viscosity (Pa·s)	Young's modulus (GPa)	Tensile strength (MPa)	Strain to failure (%)
0.5%	5	2.08 (0.2)	23 (2.2)	3.24 (0.5)
1%	5	2.06 (0.2)	22 (2.1)	3.28 (1.2)
1.5%	5	1.96 (0.3)	25 (2.5)	3.78 (0.7)
0.5%	2.5	2.9 (0.6)	48 (6.3)	4.56 (1.2)
1%	2.5	1.9 (0.4)	29 (5.8)	3.54 (0.7)
1.5%	2.5	2.6 (0.4)	30 (8.3)	4.5 (1.6)
0.5%	1.25	3.5 (0.4)	55 (5.6)	6.18 (1.9)
1%	1.25	3.3 (0.3)	51 (4.1)	8.32 (1.8)
1.5%	1.25	2.4 (0.3)	32 (6.6)	8.32 (2.2)

Table S3. Mechanical properties of solvent treated CNF epoxy composites.

CNF concentration (wt %)	Resin viscosity (Pa·s)	Young's modulus (GPa)	Tensile strength (MPa)	Strain to failure (%)
0.5%	5	1.9 (0.2)	27 (2.5)	2.8 (0.2)
1%	5	1.6 (0.1)	27 (2.6)	3.5 (0.4)
1.5%	5	1.4 (0.2)	14 (2.0)	2.02 (0.3)
0.5%	2.5	2.8 (0.2)	41 (5.0)	3.6 (0.8)
1%	2.5	2.8 (0.3)	50 (6.5)	7.1 (1.2)
1.5%	2.5	2.2 (0.1)	29 (3.0)	3.3 (0.7)
0.5%	1.25	3.4 (0.5)	60 (5.0)	6.53 (2.1)
1%	1.25	4.8 (0.4)	77 (8.0)	8.7 (1.3)
1.5%	1.25	3.7 (0.3)	61 (6.0)	7.4 (1.4)

The values in parentheses are the sample standard deviations.

© 2020 by the authors. Submitted for possible open access publication under the terms and conditions of the Creative Commons Attribution (CC BY) license (http://creativecommons.org/licenses/by/4.0/).
